# The COMET (Core Outcome Measures in Effectiveness Trials) Initiative

**DOI:** 10.1186/1745-6215-12-S1-A70

**Published:** 2011-12-13

**Authors:** Paula R Williamson, Doug G Altman, Jane M Blazeby, Mike Clarke, Elizabeth Gargon

**Affiliations:** 1 Department of Biostatistics, University of Liverpool, Liverpool, UK; 2 Centre for Statistics in Medicine, University of Oxford, Oxford, UK; 3 School of Community and Social Medicine, University of Bristol, Bristol, UK; 4 School of Medicine, Dentistry and Biomedical Sciences, Queen’s University Belfast, Belfast, UK

## Background

Systematic reviews are hampered by inconsistencies in outcomes assessed and reported in otherwise eligible studies. Many meta-analyses have to exclude key studies because relevant outcomes were not reported. Much could be gained if an agreed minimum set of appropriate and important outcomes was measured and reported in all clinical trials in a particular area.

## Why standardise outcomes?

The design of new trials would be simplified, the risk of measuring inappropriate outcomes would be reduced, and selective reporting of outcomes less likely. It would be easier to compare, contrast and combine studies in systematic reviews. Core outcome sets would help review authors to present their findings clearly and succinctly, for example within Summary of Findings tables.

## Aims of the COMET Initiative

The COMET Initiative brings together researchers interested in core outcome sets, with well attended international meetings held in 2010 and 2011. COMET aims to foster and facilitate research by providing guidance on developing a core outcome set, methods to include user involvement in this process, and preparing reporting standards for such projects. Further information about COMET can be found at [http://www.comet-initiative.org]. Work is ongoing to identify, collate and maintain relevant resources in an on-line searchable database [http://www.comet-initiative.org/studies/search]. More than 50 completed projects in various areas of health/health care have been identified. Several examples of planned and ongoing work have also been recorded. The COMET database will be demonstrated and progress to date presented.

## Implications

If successful, COMET will help trialists to choose outcomes, and will therefore increase the likelihood that these outcomes will be measured, thereby decreasing the likelihood of important studies being excluded from systematic reviews. By improving the evidence base, COMET will make it easier for people to make well-informed decisions about health care.

